# The term “supplemental parenteral nutrition” should be restricted to studies meeting specific technical criteria

**DOI:** 10.1186/s13054-017-1884-x

**Published:** 2017-12-14

**Authors:** Claude Pichard, Mette M. Berger

**Affiliations:** 1 Clinical Nutrition, Geneva University Hospital, Rue Gabrielle-Perret-Gentil 4, 1205 Geneva, Switzerland; 2 Service de Médecine Intensive Adulte et Brûlés, Lausanne University Hospital, Lausanne, Switzerland

Wischmeyer et al. recently reported the promising results of their TOP-UP pilot trial [1]. This letter aims at clarifying a semantic problem which has complicated the interpretation of many studies. The authors’ hypothesis was that supplemental parenteral nutrition (SPN) combined with enteral nutrition (EN) in ICU patients would improve 60-day survival: 125 patients on mechanical ventilation for acute respiratory failure were enrolled “within 3 days”. Patients were on EN or EN + SPN to reach 100% of the prescribed energy target, which was 25 or 20 kcal/kg actual body weight (BW) for BMI < 25 or > 35, respectively.

EN was initiated at 20 ml/h and progressively increased until the calculated energy target was reached. SPN was administered to complete the energy needs up to the energy target. Intervention was continued for 7 days.

When we proposed the SPN concept [2], the idea was to first test the patient’s tolerance to EN, as many can meet their energy needs by day 3. For those unable to reach their energy needs, SPN was proposed to cover 100% of energy needs measured by indirect calorimetry. In other words, SPN aims at rescuing situations where EN fails to cover measured needs. The beneficial impact of SPN with regards to noscomial infections in 305 patients supported the concept [3].

SPN was used in the TOP-UP trial for other reasons:

1. Additional SPN was administered within 3 days (timing not clear either) without evidence of EN intolerance.
2. Energy needs were calculated (not measured) based on actual BW, not considering fluid overload, sarcopenia, or adiposity. In patients with BMI > 35, 20 kcal/kg is likely to have resulted in serious overfeeding, a condition known to jeopardize the clinical outcome. Of note, the American Society for Parenteral and Enteral Nutrition (ASPEN) recommends 10–14 kcal/kg.
3. The full energy target was reached by day 1 in the TOP-UP group, a condition likely to result in overfeeding because of the endogenous production of energy during the early phase in the ICU [4].

In summary, the authors used the term SPN for an intervention clearly different from the original definition. Indeed, they tested an “early full feeding” strategy, potentially harmful without measurement of energy needs, instead of a delayed SPN from day 4 based on measured needs. This does not reduce the value of their results, but certainly increases confusion among the medical community. We would encourage the use of the term “supplemental parenteral nutrition” only in studies where the technical criteria of SPN are applied.

## Abbreviations

BMI: Body mass index
EN: Enteral nutrition
SPN: Supplemental parenteral nutrition

## References

[1] Wischmeyer PE, Hasselmann M, Kummerlen C (2017). A randomized trial of supplemental parenteral nutrition in underweight and overweight critically ill patients: the TOP-UP pilot trial. Crit Care.

[2] Heidegger CP, Romand JA, Treggiari MM, Pichard C (2007). Is it now time to promote mixed enteral and parenteral nutrition for the critically ill patient?. Intensive Care Med.

[3] Heidegger CP, Berger MM, Graf S (2013). Optimization of energy provision with supplemental parenteral nutrition (SPN) improves the clinical outcome of critically ill patients: a randomized controlled trial. Lancet.

[4] Oshima T, Berger MM, De Waele E (2017). Indirect calorimetry in nutritional therapy. A position paper by the ICALIC study group. Clin Nutr.

